# From Livestock to Companion: Admission Causes, Diagnostics, and Clinical Findings in Chickens Admitted to the Avian Clinic of the Vetmeduni Vienna, 2009–2019

**DOI:** 10.3390/ani15091288

**Published:** 2025-04-30

**Authors:** Cornelia Konicek, Anja Joachim, Joachim Spergser, Barbara Richter, Michaela Gumpenberger

**Affiliations:** 1 Clinical Centre for Small Animal Health and Research, Avian and Reptile Medicine, University of Veterinary Medicine Vienna, Veterinärplatz 1, 1210 Vienna, Austria; 2 Institute of Parasitology, Department of Biological Sciences and Pathophysiology, University of Veterinary Medicine Vienna, Veterinärplatz 1, 1210 Vienna, Austria; 3 Institute of Microbiology, Department of Biological Sciences and Pathobiology, University of Veterinary Medicine Vienna, Veterinärplatz 1, 1210 Vienna, Austria; 4 Institute of Pathology, Department of Biological Sciences and Pathophysiology, University of Veterinary Medicine Vienna, Veterinärplatz 1, 1210 Vienna, Austria; 5 Clinical Centre for Small Animal Health and Research, Diagnostic Imaging, University of Veterinary Medicine Vienna, Veterinärplatz 1, 1210 Vienna, Austria

**Keywords:** backyard poultry, diseases, diagnostic imaging, radiography, sonography, CT, avian, surgery, endoparasitosis, upper respiratory tract infection, crop, salpingitis, mycoplasmas, Marek’s disease

## Abstract

This retrospective study investigated the clinical presentation, diagnostic findings, and treatment outcomes in 419 backyard (pet) chickens admitted to the Service for Avian and Reptiles at the University of Veterinary Medicine Vienna from 1 May 2009 to 30 April 2019. The median age of the chickens was 1.5 years, with 83.8% being female. The majority (80.0%) presented with individual clinical issues, while 20.0% were diagnosed with flock diseases. The most common admission causes included unspecific clinical signs (57.3%), respiratory tract issues (18.4%), and locomotor problems (16.2%). Diagnostic imaging modalities such as ultrasonography, radiography, and CT scans revealed pathologies, including ascites, salpingitis, and skeletal system abnormalities. Parasitological examinations found a high rate of coccidia, *Capillaria*, and *Ascaridia*, while microbiological analysis identified *Escherichia coli* and *Pasteurella multocida* as common bacterial pathogens. The most prevalent diseases were upper respiratory tract infections (13.8%), egg peritonitis (9.9%), and soft tissue trauma (9.4%). Treatment outcomes showed that 67.5% survived to discharge, while 32.2% died or were euthanized. Surgical interventions were performed in 25.3% of cases, with the most common surgeries being salpingohysterectomy and wound debridement. This study highlights the wide range of health issues faced by pet chickens and emphasizes the importance of accurate diagnosis and targeted treatment in avian veterinary care. It also underscores the role of various diagnostic tools, such as imaging, pathogen detection, and histopathology, in addressing the health challenges of backyard chickens.

## Simple Summary

This study looked at health problems of 419 backyard (pet) chickens treated at the University of Veterinary Medicine Vienna from 1 May 2009 to 30 April 2019. The chickens were mostly young hens, averaging 1.5 years. Most animals were brought in for individual health problems; a smaller number had issues affecting the whole flock. Common reasons for visits included general sickness (such as sleepiness and poor appetite), breathing difficulties, and mobility problems. To diagnose the issues, veterinarians used various tests, including imaging techniques like sonography, radiography, and CT scans. These revealed several conditions, such as fluid buildup, reproductive infections, and broken bones. Many chickens also had parasites, such as coccidia and roundworms, as well as bacterial infections caused by *Escherichia coli* and *Pasteurella multocida*. The most common health issues included respiratory infections, diseases related to egg production, and injuries. In terms of treatment outcomes, around two-thirds of the chickens survived and were discharged, while one-third either died or were euthanized. Some chickens needed surgery, like removal of infected reproductive organs or treatment for wounds. This study highlights the variety of health problems pet chickens can face and emphasizes the importance of accurate diagnosis and treatment. It also shows how crucial diagnostic tools, such as imaging methods and laboratory tests, are in identifying and addressing these health issues. Understanding common conditions and providing proper treatment is indispensable to ensure the well-being of this special group of pet animals.

## 1. Introduction

Keeping chickens in urban or suburban neighborhoods has become increasingly popular in recent years, not only in Europe but also in Canada, the United States, and Australia [1,2,3,4]. These chickens are widely designated as “backyard chickens”. Most of them have free-range access or an enclosed run during the daytime [5]. Still, many animals are not strictly confined to the outdoors; therefore, the term backyard chickens might not be completely accurate [4]. Furthermore, backyard chickens, especially in resource-poor tropical countries, are kept as a food source and not, like in industrialized countries, as pets, and therefore usually do not receive that level of attention and care. The term non-commercial chicken might be a more suitable term [4]; still here, we decided to use the terms “backyard” and “pet” chicken, as these are currently most commonly used for such chicken populations. The motivation for keeping these chickens includes not only the consumption of their eggs but also their use as gardening partners (pest control and manure as fertilizer) and as pets [5]. Unlike the well-controlled and regulated commercial poultry flocks, backyard poultry is typically only presented to the veterinarian in cases of clinical signs or disease outbreaks. Owners often seek veterinary care for individual pet chickens at small or exotic animal clinics, which can present challenges for the attending veterinarian. Treating an individual diseased chicken differs significantly from managing flock-wide diseases, requiring a different approach to diagnosis and care. Moreover, chicken is classified as a food animal species by law, and, accordingly, veterinary care for pet chickens has limitations in the use of licensed medication, at least in the European Union. Additionally, owners frequently lack basic knowledge of flock management, general hygiene, and biosecurity protocols. The chickens often come from various sources and have not received prophylaxis against infectious diseases such as vaccination, which can lead to disease transmission and pathogen circulation within the flocks. Few studies have focused on common mortalities and health status in pet chickens in previous years [2,6,7,8,9]. Still, research providing insights into health issues, especially in urban and suburban backyard chicken flocks, is scarce. While some individual studies have focused on emergency presentations and postmortem investigations, the main health issues of pet chickens presented to veterinarian practitioners and clinics have not been quantified.

Most small animal veterinarians have limited experience with poultry [10], yet there is an increasing demand from clients for individual medical care for their pet chickens. Consequently, retrospective data from an avian clinic in Vienna, Austria, were analyzed to provide insights into common problems, discussing the challenges of treating pet chickens in clinical practice. This analysis focused on the causes of pet chicken admissions, diagnostics, clinical findings, and outcomes of backyard chickens presented over a decade, from 2009 to 2019.

## 2. Materials and Methods

The electronic medical records of backyard poultry presented at the Service for Avian and Reptile Medicine, Clinical Unit for Small Animals, University of Veterinary Medicine in Vienna, were searched for cases from 1 May 2009, to 30 April 2019. The records were subsequently retrieved and reviewed, and the relevant information was collected. This included data on breed, gender, age, whether the case was an emergency or a routine visit, the primary medical conditions, and whether the problem affected the individual bird or the flock. The diagnostic modalities used were noted, including hematology, diagnostic imaging, direct and indirect pathogen detection, necropsy, and histopathology results. The final confirmed or provisional diagnosis, selected treatment, and outcome (when available) were also recorded.

On each admission, owners were informed about the costs of necessary diagnostic procedures and treatment options to ensure compliance. In addition, owners were required to sign a consent form acknowledging that their chickens would be treated as pets. If medications not licensed for food animals were used, owners were informed that their chickens’ eggs and meat should no longer be consumed.

Admission causes were defined based on the clinical signs observed by the owners; each case had one or more admission causes assigned. These signs were further grouped according to the affected organ system. Physical examinations, sampling, and treatments were conducted by avian specialists. Advanced diagnostic procedures were performed by specialized clinics and institutes at the University of Veterinary Medicine Vienna, including the Clinic of Diagnostic Imaging, the Clinic for Poultry and Fish Medicine, and the Institutes of Microbiology, Pathology, and Parasitology.

Primary diseases and clinical signs were categorized by the affected organ system, including the integument, locomotor system, ophthalmologic system, neurological system, respiratory tract, cardiovascular system, reproductive tract, gastrointestinal tract, hepatic system, systemic conditions, or unspecified alterations.

Whenever more than one bird from a flock presented similar clinical signs, or the owner reported that multiple birds appeared to be affected, the case was classified as a flock disease. Treatment was based on the clinical findings of the birds that were presented. However, no flock visits were conducted, and specific data regarding flock size or the age of birds not presented were not collected.

Treatments were classified as inpatient or outpatient and further categorized as involving licensed medications, non-licensed medications, surgery, conservative methods, or no treatment.

Descriptive statistics were computed using Microsoft Excel 365.

### 2.1. Diagnostic Modalities

This manuscript presents a retrospective analysis of the admission causes, diagnostic procedures, and clinical findings in chickens examined and treated at a specialized avian medicine service within the Clinical Centre for Small Animal Health and Research at the University of Veterinary Medicine, Vienna. Standardized experimental or diagnostic protocols were not uniformly applied across all cases; instead, diagnostic approaches were guided by clinical indications and owner consent. An overview of the commonly employed diagnostic protocols is provided in the following subsections.

#### 2.1.1. Diagnostic Imaging

Radiographs were obtained using a mobile PX 20 unit (Poo Yee X-Ray, New Taipei City, Taiwan) with imaging plates processed by the FCR Capsula system (CR-IR 357, Fujifilm Holdings Corporation, Minato, Tokyo, Japan). Imaging was performed either on awake birds or under isoflurane anesthesia. Standard radiographic views were taken when indicated. In critical patients where radiodense eggs or foreign bodies were suspected, only survey radiographs in dorsoventral projection were performed.

CT scans were performed with a 16-slice helical CT, Siemens Somatom Emotion (Siemens, Vienna, Austria), using 80–100 mAs, 130 kV, rotation time 1.5 s, pitch 0.8, and slice thickness of 0.5 to 0.75 mm. The chickens were placed in sternal recumbency or in the upright standing position without any sedation in closed cupboards or plastic boxes. The scans were reformatted with an ultrasharp bony and a soft tissue kernel, matrix size of 512 × 512, increment of 0.6 mm, and evaluated in a bony and soft tissue window. Image interpretation was performed with JIVEX, Version 5.3.0.2 RC01 (Visus Health IT GmbH, Bochum, Germany).

Sonographic examinations took place with patients in the upright standing position with barely any manual restraint. In several accessible windows, the feathers were parted and the skin of the abdomen moisturized with some gel. The examinations were performed using a Phillips unit (iU22 Ultrasound System, Phillips, Bothell, WA, USA) and a 5–8 MHz microconvex transducer.

#### 2.1.2. Hematology

For hematological examinations, blood was collected from either the vena basilica, the medial vena metatarsalis, or the right vena jugularis using a heparinized syringe and needle. Samples were immediately sent to the laboratory of the University of Veterinary Medicine Vienna and processed within a few hours according to standard diagnostic procedures.

#### 2.1.3. Microbiological Examinations

When clinically indicated and with the owner’s consent, sterile cotton-tipped swabs with transport medium (CliniswabTS, APTACA S.p.A., Canelli, Italy) were collected for microbiological examination. Depending on the clinical signs, samples were taken from the conjunctiva, nares, choana, pharynx, trachea, crop, cloaca, feces, wounds, abscesses, synovial fluid, or internal organs such as the intestinal tract, liver, and serosa.

For bacteriological examination, samples were plated onto a general-purpose agar (Columbia III agar with 5% sheep blood, BD Diagnostics, Vienna, Austria) and various selective and differential agars depending on sample type (e.g., MacConkey II agar, Colistin-Nalidixic Acid agar, Xylose-Lysine-Desoxycholate agar, Campylobacter blood-free agar) (all BD Diagnostics, Vienna, Austria) using the three-phase streaking method. Agar plates were incubated aerobically, microaerobically, or anaerobically at 37 °C for 48 h. For mycological examination, samples were plated onto Sabouraud agar with gentamicin and chloramphenicol (BD Diagnostics, Vienna, Austria) and incubated at 28 °C for up to 7 d. Microbial isolates were identified using standard identification methods such as biochemical tests (API^®^, BioMérieux, Vienna, Austria) (before 2016) or by matrix-assisted laser desorption ionization-time of flight (MALDI-ToF) mass spectrometry (Biotyper^®^, Bruker Daltonics, Bremen, Germany) (from 2016 on), as described previously [11]. For the cultural detection of avian mycoplasmas, samples were cultivated on FREY agar (Millipore, Merck, Vienna, Austria) and incubated at 37 °C under a 6% CO_2_ atmosphere for up to 10 days. Mycoplasma colonies were identified to the species level using standard 16S rRNA gene sequencing (before 2016) or MALDI-ToF mass spectrometry, as described earlier [12].

#### 2.1.4. Wet Mount Preparations

Whenever indicated, wet mounts of crop and fecal samples were prepared and immediately examined using a light microscope with 4×, 10×, and 40× objectives (Nikon, Vienna, Austria), with particular attention to the presence of *Candida*, flagellates, and other parasitic organisms.

#### 2.1.5. Parasitological Coproscopy

For qualitative parasitological coproscopy, flotation in combination with sedimentation was carried out. For this, the entire fecal samples were mixed with approximately 10 mL of tap water using a mortar and pestle and sedimented by centrifugation in a 14 mL round-bottom tube at 690× *g* for 8 min in a table centrifuge with swing-out rotor (Universal 16, Hettich, Tuttlingen, Germany). The supernatant was decanted, and the sediment was resuspended in approximately 3 mL of flotation solution (saturated sucrose solution, specific gravity at 26 °C = 1.28) using a minishaker (IKA, Staufen, Germany) for 3 × 10 s. The flotation solution was topped up to ca. 5 mm below the rim of the tube, and the sample was centrifuged at 690× *g* for 8 min. Then the surface liquid, including any floated eggs/oocysts, was removed using a metal loop (approx. 5 mm diameter), transferred to a glass slide, covered with a glass coverslip, and examined under light microscopy (Nikon, Vienna, Austria) at 100× magnification. For exact determination of parasite stages, higher magnifications (200× or 400×) were used. The complete area under the slip was examined.

#### 2.1.6. Cytology

In a few cases, fine needle aspiration biopsies, synovial fluid, or coelomic fluid samples were submitted to the routine diagnostic laboratory of the University of Veterinary Medicine Vienna for further examination.

#### 2.1.7. Serology

In a few cases, whole blood samples were sent to the Clinical Centre for Population Medicine in Fish, Pig, and Poultry at the University of Veterinary Medicine for serological testing to detect antibodies against infectious laryngotracheitis (ILT) virus, infectious bronchitis (IB) virus, avian rhinotracheitis virus, *Ornithobacterium rhinotracheale*, duck adenovirus 1 virus (egg drop syndrome), *Mycoplasma gallisepticum*, and *Mycoplasma synoviae*, following their routine diagnostic procedures.

#### 2.1.8. Necropsy and Histopathology

Whenever accepted by the owner, gross pathological and histopathological examinations were performed on deceased or euthanized birds. For histopathological examination, tissue samples were fixed in 4% neutral buffered formaldehyde for at least 24 h. After trimming, the samples were embedded in paraffin wax. Sections with a thickness of 3 µm were mounted on glass slides and stained with hematoxylin and eosin (HE) following standard protocols. A coverslip was mounted on top, and the slides were examined using light microscopes with 2.5, 10, 20, and 40× objectives.

## 3. Results

### 3.1. Cases

**Figure 1:**
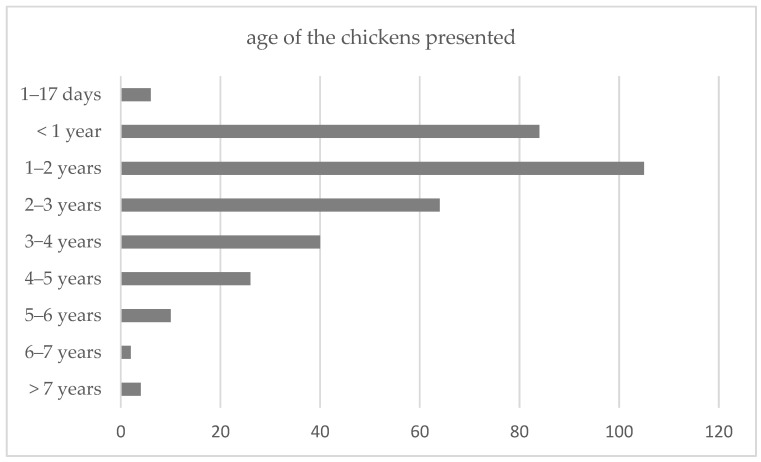
Age distribution of the chickens presented during the study period. The median age was 1.5 years.

A total of 419 individual chickens (*Gallus gallus domesticus*) were admitted to the Service for Avian and Reptiles of the University of Veterinary Medicine Vienna during the observed decade. There was a general increase in the number of annual cases. On average, there were 39 chicken cases per year, ranging from a minimum of 8 to a maximum of 72 cases. The median age of the chickens was 1.5 years, with a range from 1.4 days to 11 years (Figure 1).

The majority (83.8%) of the chickens were female, whereas roosters were presented as a minority (13.1%). In 13 cases (3.1%), the sex of the chickens has not been recorded.

A specific breed was noted in 97 cases (23.3%), which included Bantam (11.0% from 419 chickens presented), Silkie (2.6%), Sulmtaler (1.7%), and Orpington (1.0%). Breeds represented in less than 1% of cases included Amrock, Araucana, Königsberger, Leghorn, Braekel, Padovana, Appenzeller Spitzhaube, Easter Egger, Bielefelder Kennhuhn, Brahma, Barnevelder, and Alsteirer. The majority of chickens (76.8%) did not have a specific breed recorded, likely representing egg-laying hybrids.

A significant proportion (28.6%) were presented as emergency cases, and the majority (62.5%) required in-clinic treatment. Out of the cases presented, 20.0% were classified as flock diseases, while the majority (80.0%) involved clinical issues in individual chickens.

### 3.2. Admission Causes

Admission causes were given in 386 of the cases, while for the remaining 33 cases, the information in the records was missing (Table 1). The most common reasons for admission were attributed to unspecific clinical signs (57.3%), including lethargy (30.3%), anorexia (11.0%), distended coelom (8.8%), emaciation (4.1%), and mortality events (3.1%). The second most common admission causes were associated with the respiratory tract (18.4%), including respiratory distress (11.7%) and nasal discharge (6.7%) (Table 1).

### 3.3. Applied Diagnostic Modalities

Further diagnostic workup was applied whenever indicated and accepted by the owner, including diagnostic imaging, hematology, blood chemistry, direct and indirect detection of pathogens (including serology, cytological, microbiological, and/or parasitological examinations of specimens), gross necropsy, and histopathology of organs or biopsy samples. A table summarizing the diagnostic modalities used, grouped by admission causes, can be found in Supplementary Table S1.

#### 3.3.1. Diagnostic Imaging

Out of 130 diagnostic imaging examinations, 98 chickens underwent coelomic ultrasonography, 30 whole-body radiography, and in 26 cases, a computed tomography (CT) scan was performed. One or more diagnoses were assigned to the respective diagnostic imaging modalities. The most common diagnoses made by ultrasonography were ascites (34.7%), followed by malformed eggs (17.4%) and salpingitis (17.4%). Radiography detected most often pathologies of the skeletal system (26.7%) and salpingomegaly (6.7%). With the help of CT, the most common finding was salpingitis (30.8%), followed by pathologies of the skeletal system (23.1%) (Table 2).

In 107 cases, only a single diagnostic imaging modality was used. Among these, ultrasonography was the most frequently applied technique (76 cases), followed by radiography (16 cases) and CT (15 cases). In 21 cases, two imaging modalities were used as follows: radiography and ultrasonography (12 cases), ultrasonography and CT (8 cases), and CT and radiography (1 case). Additional imaging techniques were employed for various reasons, including reproductive tract pathologies (8 cases), distended coelom initially assessed with survey radiograph to rule out calcified eggs or radiodense egg material (5 cases), gastrointestinal tract pathologies (4 cases), soft tissue masses (2 cases), hepatopathy (1 case), and respiratory tract pathologies (1 case). In two cases, all three imaging modalities were used. Both chickens presented with multiple soft tissue masses in the coelom, with one ultimately diagnosed with Marek’s disease and the other with carcinomatosis.

#### 3.3.2. Parasitological Examination

Fecal flotation and copromicroscopy were carried out in 151 patients. Of those, 66.2% were positive, with 38.4% of samples positive for a single parasite and 27.8% positive for two or more. One-third of the samples (33.8%) were positive for oocysts of coccidia, followed by eggs of *Capillaria* (29.8%) and *Ascaridia* (19.2%). Less frequently, *Heterakis* (6.0%), cestodes (*Raillietina* or *Hymenolepis*) (4.0%), motile protozoal parasites (3.3%), and *Trichostrongylus* (2.0%) were detected. No parasites were detected in 33.8% of the samples.

Clinical signs that could be attributed to endoparasites were seen in 31 of the cases. Most presented anorectic and lethargic (14 cases), seven were emaciated, six had diarrhea, three had cloacal prolapse, two shed partially undigested food, and two had crop stasis.

Shedding oocysts of coccidia was most frequently observed in chickens <1 year (43.9% of the samples positive for coccidia), with the next highest occurrence in animals aged 1–2 years (34.1%).

#### 3.3.3. Microbiological Examination

In total, 87 samples were microbiologically examined, including antibiotic susceptibility testing whenever possible. Samples were taken from one or more regions: nose, conjunctiva, choana, trachea, pharynx, crop, cloaca, wounds, fluids, or inner organs. The most commonly isolated agents were *Escherichia* (*E*.) *coli* (47.1%), followed by *Pasteurella multocida* (21.8%), and coagulase-negative *Staphylococcus* spp. (17.2%). Less frequently isolated microbes were *Streptococcus* spp. (9.2%), *Enterococcus* spp. (8.0%), and *Pseudomonas* spp., as well as *Gallibacterium anatis* and *Candida albicans* (6.9% each) (Table 3).

Six samples were negative for bacterial or fungal growth. Of these, samples were harvested from a dilated crop, from a liver with mixed-cell pericholangitis of unknown origin, two from the conjunctiva with signs of conjunctivitis and sinusitis, and two samples from the crop, choana, and cloaca due to unspecific signs.

Samples from choana and nares were taken for specific detection of mycoplasmas in 35 chickens. Of those, 18 were positive for less pathogenic *Mycoplasma* species; 11 samples were positive for pathogenic *Mycoplasma* species, namely, *M. gallisepticum* (5) and *M. synoviae* (6); 10 samples were negative for mycoplasmas. In addition, serological tests for mycoplasma antibodies were carried out on another 8 chickens. Of those, high titers of antibodies against *M. gallisepticum* were found in five chickens, and antibodies against *M. synoviae* in three chickens. None of the chickens were vaccinated. Most birds that tested positive for mycoplasmas exhibited clinical signs of upper respiratory tract infection, while two individual chickens showed symptoms associated with lower respiratory tract infections (Table 4).

### 3.4. Affected Organ Systems

**Figure 2:**
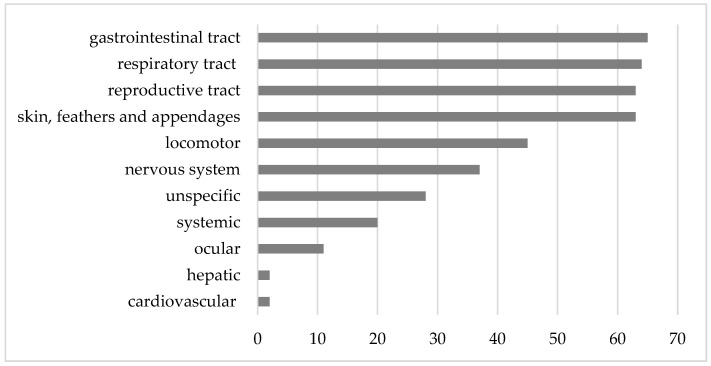
Total numbers of cases according to affected organ systems within the study period.

Based on the recorded data of anamnesis, clinical symptoms, and findings of the applied diagnostic modalities, a primary disease was assigned and categorized by the underlying organ systems. The most common diseases were associated with the gastrointestinal tract (15.5%); the respiratory tract (15.3%); skin, feathers, and appendages (15.0%); and the reproductive tract (15.0%); followed by locomotor diseases (10.7%) and diseases of the nervous system (8.8%) (Figure 2).

Only one organ system was involved in 338 cases, two organ systems in 39 cases, and three in three cases. If more than three organ systems were affected, the category “systemic diseases” (4.8%) was selected.

#### 3.4.1. Gastrointestinal Tract

Regarding diseases of the gastrointestinal tract (65 cases), the majority were attributed to endoparasites (44.6%). One or up to 4 of the following endoparasites were linked to diseases of the gastrointestinal tract: *Capillaria* (58.6%), coccidia (48.3%), *Ascaridia* (48.3%), motile protozoal parasites (13.8%), *Heterakis* (10.3%), cestodes (10.3%), and *Raillietina* or *Hymenolepis* and *Trichostrongylus* (3.4%).

The second most common issue of the gastrointestinal tract was related to the crop (18.5%). Among the 12 crop pathologies, 6 cases involved bezoars, 2 cases featured crop dilatation, 2 cases exhibited crop stasis, and one was associated with bacterial ingluvitis.

Other diseases categorized under gastrointestinal tract disorders included bacterial gastroenteritis (13.8%), candidiasis (7.7%), neoplastic diseases (6.2%), and cloacal prolapse (6.2%). One case had an obstipation, and one case had Marek’s disease (MD), which was linked to proventriculus infiltration.

The majority of cases with gastrointestinal tract diseases were 1–2 years old (30.8%), followed by 2–3 years (20.0%), <1 year (16.9%), 3–4 years (13.8%), >4 years (10.8%), and unknown (7.7%).

#### 3.4.2. Respiratory Tract

Out of the 64 pathologies associated with the respiratory tract, 79.7% were related to the upper respiratory tract and 20.3% pertained to the lower respiratory tract. Most of the chickens with respiratory tract diseases were <1 year old (14.1%), followed by unknown age (7.7%), 1–2 years (9.6%), 2–3 years (4.5%), 3–4 years (3.8%), and >4 years (1.3%).

Among these cases presenting clinical signs of upper respiratory tract infections, microbiological examinations were conducted in only 28 instances, with 27 specifically tested for mycoplasmas. The most frequently isolated bacterial pathogen was *E. coli*, followed by *Pasteurella multocida* (Table 5).

Among these cases, 15 revealed the isolation of a single bacterial pathogen, while 11 cases presented mixed cultures. Out of the 25 chickens that tested positive for mycoplasmas, 11 were infected by well-known pathogenic *Mycoplasma* spp. (i.e., *M. gallisepticum*, *M. synoviae*). In four cases, more than one *Mycoplasma* species was detected, and in 18 cases other bacteria were isolated in addition to mycoplasmas (Table 4).

The definitive cause of the lower respiratory tract diseases was not determined in 8 cases. Three cases showed high antibody titers against infectious bronchitis (IB) virus, one case was associated with infectious laryngotracheitis (ILT) virus, and one case was attributed to trauma.

#### 3.4.3. Reproductive Tract

The majority of the reproductive tract pathologies (63 cases in total) were egg yolk peritonitis, salpingitis, or salpinx impaction (48.4%). This was followed by neoplasia of the reproductive tract (21.0%). Less frequently, cases were associated with dystocia of a physiological shelled egg (11.3%), cloacal prolapse linked to egg laying (8.1%), four cases involved shelled eggs or egg material freely present in the coelom, two cases featured soft-shelled eggs as the sole finding, and one case exhibited a cyst associated with the salpinx. The ages of chickens with reproductive tract pathologies were documented in 49 cases. The diagnosis was most often identified in the 2–3 years old category (24.2%), followed by chickens with unknown age (21%). The frequency of reproductive tract diseases was the same in the 1–2 year and 4–5 year age groups (Table 6).

#### 3.4.4. Skin, Feathers, and Appendages

Integument pathologies (63 cases) encompassed a range of conditions, with trau-matic skin wounds being the most common (47.6%), followed by ectoparasites (23.8%), pododermatitis (20.6%), issues related to poor feather quality and dermatitis of undefined origin (6.3%), and beak deformities (1.6%). Among the ectoparasites, eight chickens had a manifestation with scaly leg mites (*Knemidocoptes gallinae*), three had poultry red mites (*Dermanyssus gallinae*), two had heavy infestations with chewing lice, and two had feather mites. One chicken was infested with three different ectoparasites, while one was infested with two different ectoparasites.

Regarding cases with pathologies of skin, feathers, and appendages, most chickens were in the 1–2 year age group (31.7%), followed by those of unknown age (22.2%), <1 year (19.5%), 2–3 years (12.7%), 3–4 years (9.5%), and >4 years (4.8%).

#### 3.4.5. Locomotor System

Orthopedic issues (45 cases) were diverse and included malformations (31.1%), fractures (20.0%), joint diseases such as arthritis and arthrosis (17.8%), unexplained causes of lameness (15.6%), and soft tissue traumas (11.1%), along with one case of neoplasia and one case of femoral head necrosis.

Most orthopedic cases were presented in chickens less than 1 year old (37.8%), followed by those in the 1–2 year age group (22.2%), unknown age (22.2%), 3–4 years (13.3%), 2–3 years (6.7%), and >4 years (4.4%).

#### 3.4.6. Nervous System

Regarding central nervous system disorders (37 cases), the majority had suspicion of MD (62.2%), with confirmation through necropsy and histopathology in 10 instances (27.0%). Additionally, three cases were confirmed to have encephalitis based on necropsy and histopathology, and one chicken exhibited torticollis of unknown origin.

Of the cases with nervous system diseases, the majority were less than 1 year old (35.1%), followed by unknown (24.3%), 1–2 years (18.9%), 2–3 years (13.5%), and >3 years (8.1%).

### 3.5. Final Diagnosis and Final Outcome

A final diagnosis was assigned in 363 cases. Around one-third of the animals improved or recovered after treatment, one-third died or had to be euthanized, and one-third were lost to follow-up. Diagnosis, treatment, and outcome are summarized in Table 7.

The most common disease was upper respiratory tract infection (13.8%), followed by egg yolk peritonitis (9.9%) and soft tissue trauma (9.4%). When considering orthopedic traumatic cases, the total observed trauma cases amounted to 43 (11.8%). Among cases with identifiable traumatic events, the occurrences were evenly distributed among bird attacks, companion chickens, other pets (dog/cat), and predator attacks (mostly from mustelids), each accounting for three cases, while one case was related to technopathy.

In the group of the neoplastic cases (7.2%), the most common diagnosis was carcinomatosis (73.1%), characterized by metastatic adenocarcinomas originating from either the reproductive tract or the pancreas. Notably, one case was linked to MD, and another exhibited secondary neoplasia—a leiomyoma of the salpinx. Two instances involved masses associated with the reproductive tract with undefined characteristics. Furthermore, one case featured a squamous cell carcinoma within the pharynx. One chicken presented with neoplasia in the kidney, accompanied by metastasis in the lungs. Another case involved a mass in the left shoulder extending to the bones, with metastasis observed in the vertebrae, femora, and synsacrum. Additionally, two cases manifested masses originating from the infraorbital sinus, one diagnosed as lymphoma and the other as schwannoma. In terms of age, records were available for 21 cases. Two cases were less than one year old, while the remaining 19 were older than two years.

### 3.6. Treatment

A total of 262 chickens were hospitalized and received treatment at clinics, while 157 chickens were treated as outpatients at home. Treatments included the use of licensed medication in 119 cases, while non-licensed medication was required for 236 individual cases. A total of 92 chickens underwent surgical procedures, with the most common being surgery of the reproductive tract, such as salpingohysterectomy (26.1%), followed by wound debridement (19.6%), laparotomy (14.1%), debriding of pododermatitis lesions (10.9%), and repositioning of cloacal prolapses and ingluviotomy (7.6% each). Additionally, various other surgical interventions were undertaken (Table 8).

Eighteen chickens received conservative treatments involving bandages for malformations (splayed legs) (5), fractures (4), pododermatitis (4), crop bandages (2), and various other conditions like skin wounds, luxation of the phalanx, and tendon lesion (1 case each). In 87 cases, no treatment was implemented, either because the owner declined treatment or because no therapy was deemed necessary. In 239 cases, the off-label use of medication was noted. These medications were categorized into antibiotics, analgesics, anesthetics, antifungals, antiparasitics, and others (Table 9).

### 3.7. Outcome

Among all 419 cases presented, 31.7% showed improvement, with their clinical signs resolving. In 35.8%, there was no follow-up visit, and information about their progress was unavailable, although they survived to discharge. Additionally, 18.4% of chickens were euthanized, and 13.8% succumbed to their conditions. In summary, 283 out of 419 cases (67.5%) survived to discharge, while 135 out of 419 cases (32.2%) died or were euthanized.

## 4. Discussion

### 4.1. Importance of This Study

Diseases commonly observed in commercial poultry can also affect pet chickens. However, differences in lifestyle and biosecurity management between these settings lead to variations in disease prevalence. Pet chickens, which typically have outdoor access and are kept in small flocks, often lack routine health checks and disease control plans. These chickens are commonly owned as hobby animals by individuals who may not be fully aware of biosecurity measures or key infectious diseases [5,13].

Preventive medicine holds a significant role in commercial poultry flocks, but this is not typically the case for backyard poultry. This is evident in the markedly low frequency of general health checks seen in our study (1.7% of the presented animals), compared to the high number of emergency presentations (28.6%) among cases brought to the clinic. Similar trends have been observed by other researchers [4,9,14,15]. This is likely due to the inherent stoic nature of chickens, making it challenging for pet owners to detect early signs of illness. Consequently, these chickens are often assessed for conditions that have already advanced, requiring urgent care [15].

Additionally, while commercial chickens rarely live beyond 1–2 years, backyard chickens can live for 11 years and longer [4]. This aligns with the current findings, as the oldest chicken presented to the clinic was 11 years old, while the median age at presentation was 1.5 years—a trend also observed in previous studies [2,3,15].

Many backyard chicken owners seek veterinary care at general small-animal veterinary practices that accommodate chickens [4]. However, these clinics often have limited experience with poultry or avian species in general. With increasing numbers of chickens being kept as pets, the need for specialized veterinary services that address not only common health problems but also offer preventive care, diagnostics, and tailored treatments is essential. Gaining an understanding of the most common diseases affecting backyard chickens can therefore be highly beneficial. Currently, data on common health issues in backyard chickens are primarily available through studies on emergency presentations [3,15] or postmortem examinations [2,6,7].

In contrast, this study provides an overview of common complaints and diseases seen in backyard chickens during routine visits to small-animal and exotic practices. Consequently, it offers valuable insights to both general and exotic animal veterinarians, enhancing their ability to manage backyard chicken cases in daily practice.

### 4.2. Cases

While keeping backyard chickens has been popular since the inception of this study, owners previously showed less inclination to seek veterinary care for individual chickens [13,16]. In contrast, a recent survey found that over 63% of chicken owners took their sick chickens to a veterinary practice [4]. This aligns with the observed increase in the number of backyard chickens seen in this study. However, whether this trend reflects greater willingness among owners to seek veterinary care or simply an increase in the number of backyard chickens could not be determined in this survey.

At the beginning of each consultation, the purpose of the chicken was discussed with the owner. Treating individual diseases in chickens often requires costly diagnostic tools and treatments, along with the use of non-licensed medications [14]. Therefore, we consider these chickens as pets rather than food-producing animals. Additionally, owners are required to sign a special consent form whenever off-license medication is prescribed. Most owners prioritize their chicken’s well-being over egg production, reflecting a shift toward viewing backyard chickens as pets or companion animals rather than livestock—a trend noted in previous studies as well [4,5,17]. This emotional bond should be considered when providing care for pet chickens. Our findings reflect this, as owners in this study were often willing to accept costly diagnostic imaging, such as CT scans (6.2% of cases), surgical interventions (22.0%), and hospitalization (62.5%).

### 4.3. Admission Causes

Most of the chickens were brought in due to nonspecific clinical signs, notably lethargy (30.3%). Previous studies also highlighted a high prevalence of chickens presented with nonspecific signs of illness [9,15]. This underscores the necessity for comprehensive anamnesis, clinical examination, and further diagnostic investigation.

### 4.4. Diagnostic Imaging

This study demonstrates the importance of diagnostic imaging (ultrasonography, radiography, and CT scans) in detecting various internal pathologies, such as ascites, salpingitis, and skeletal abnormalities. In many cases, more than one imaging modality was necessary for an accurate diagnosis. When only a single technique was used, ultrasonography was the most frequently applied. However, in some cases, ultrasonography alone was insufficient due to limited coupling sites, image noise, or artifacts caused by intestinal contents or the air sac system.

While these methods are associated with increased costs, they reflect the willingness of pet chicken owners to provide advanced veterinary care for their animals. Diagnostic imaging enables veterinarians to identify conditions that may not be visible during a physical examination, leading to more accurate diagnoses and more effective treatments.

Within this survey, radiography proved to be most useful for diagnosing skeletal pathologies. Ultrasonography was especially helpful for detecting ascites, as well as malformed eggs and salpingitis or liver disease. CT scans provided the most comprehensive diagnostic information, as they allow for detailed evaluation of multiple organ systems beyond what radiography and ultrasonography alone can offer. It may be classified as the gold standard for the evaluation of the respiratory tract. However, among the diagnoses made with the help of CT, salpingitis was the most common.

### 4.5. Assigned Organ Systems and Common Diseases

While post-mortem studies revealed infectious diseases as the leading cause of mortality [1], particularly MD and *E. coli* septicemia [2,6,7,8], studies focusing on emergency visits identified trauma cases [15] and reproductive tract diseases as the most common issues [3]. The data from this survey indicated a diverse range of problems, with relatively even distributions among pathologies associated with the gastrointestinal tract (15.5%), respiratory tract (15.3%), reproductive tract (15.0%), and skin, feathers, and appendages (15.0%).

#### 4.5.1. Gastrointestinal Tract

The incidence of diseases or clinical signs associated with the gastrointestinal tract was notably high (15.5%) in this study, with most cases linked to endoparasites. An extensive survey of non-commercial chicken owners in the United Kingdom identified diarrhea as the second most common health issue after red mite infestations [4]. In contrast, only 31.2% of these owners reported always deworming their chickens or routinely sending fecal samples for testing. Alarmingly, 24.4% stated that they never perform these preventative measures [4].

Within this survey, cases attributed to endoparasites were predominantly associated with roundworms, namely, *Capillaria* and *Ascaridia*. Followed by coccidia, particularly in chickens younger than 1 year. Findings from other studies identified coccidiosis as the most common endoparasitic infection in backyard chickens [1,2,6]. However, the true prevalence of endoparasitic infections in backyard chickens could not be determined in this study, as not all chickens underwent fecal examination. There is likely a significant bias toward cases presenting with clinical signs attributed to gastrointestinal disease.

Interestingly, gastrointestinal parasites were mostly not associated with clinical signs. Notably, 58% of positive fecal samples had only one detected parasite, while 42% were positive for two or more parasites. The presence of multiple parasites in positive fecal samples was expected, given the typically extensive husbandry practices in backyard poultry. To mitigate the risks of endoparasitic infections, regular check-ups—ideally every six months—with fecal samples collected from the entire flock are recommended. This approach ensures accurate diagnosis and treatment, when necessary, while also facilitating environmental control measures. Such measures include rotating pens, maintaining low population densities, frequently removing droppings, ensuring runs are well-drained, allowing droppings and soil to dry, providing good ventilation, and ensuring access to ultraviolet light [17].

The second most prevalent findings associated with the gastrointestinal tract were pathologies of the crop, primarily presenting as crop impactions with grass bezoars, all of which necessitated surgical intervention. Generally, crop abnormalities like delayed passage or complete stasis often signal underlying gastrointestinal or systemic diseases [15]. Hence, such cases should always undergo comprehensive diagnostic workups.

The prevalence of cloacal prolapse was relatively low, accounting for 2.4% (10/419), with the majority presenting as emergencies (7/10). A slightly higher prevalence of 3.6% among emergency presentations only was documented in a previous study [3], which exhibited a notably poor outcome for these cases, with a survival rate of only 25%. Conversely, in this current study, the observed cases generally had a favorable outcome, with 7 cases (70%) resulting in recovery, only one case leading to death, and two cases lost for follow-up. Overall, five cloacal prolapses were attributed to issues in the reproductive tract, four to gastrointestinal tract problems, and in one case the cause remained unidentified. Cloacal prolapse should always be considered as an emergency presentation [3], necessitating immediate veterinary care and comprehensive examinations to identify the underlying causes of the condition. After emergency prolapse repositioning, diagnostic measures such as digital cloacal palpation, diagnostic imaging, and at least parasitological examination of feces are essential in these cases.

#### 4.5.2. Respiratory Tract

In cases involving respiratory tract diseases, the highest proportion was attributed to upper respiratory tract infections. Further examinations of these cases unveiled the identification of various agents. Among these, *E. coli* and mycoplasmas stood out as the most associated bacterial agents, although various bacteria were isolated.

Mycoplasmosis denotes a contagious disease prevalent worldwide affecting the respiratory, musculoskeletal, and reproductive tracts of poultry [18]. Control measures and serology-based surveillance programs in commercial poultry flocks across many countries have successfully eradicated pathogenic *Mycoplasma* species from most chicken breeder flocks. However, this does not apply to backyard chicken breeders and flocks, where mycoplasmosis appears to be the most common respiratory condition [7].

In this survey, a total of 35 chickens were tested for mycoplasmas, with 25 showing positive results. Among them, the majority were associated with upper respiratory tract infections. Notably, five of these chickens tested positive for *M. gallisepticum*, a pathogen known to cause chronic respiratory disease and significant economic losses in poultry production. This finding suggests that backyard flocks could serve as reservoirs for *M. gallisepticum*, posing a potential risk of spillover into commercial operations. Although most of these chickens were colonized by mycoplasmas with no or little evidence of pathogenicity (apathogenic/opportunistic *Mycoplasma* species), many presented concurrent bacterial infections, suggesting the potential involvement of these mycoplasmas in the ongoing infections. This underscores the significance, especially in cases suspicious of respiratory tract infections, not only of conducting regular microbiological cultures but also specifically testing for mycoplasmas.

*Escherichia coli* is widely recognized for its involvement in various infections and is considered a significant cause of mortality in backyard poultry [2,6,7]. In this study, *E. coli* was the most common cause of upper respiratory tract infection. The second most common isolate was *P. multocida*, the causative agent of fowl cholera, a highly contagious disease responsible for significant mortality and economic losses in commercial poultry. Low detection rates of *P. multocida* in backyard flocks have been previously described [2,6,7]. This finding suggests that while *P. multocida* remains a pathogen of concern, its prevalence in backyard chickens may be sporadic, warranting further investigation into the ecological and epidemiological factors influencing its occurrence. Importantly, outbreaks of fowl cholera are notifiable diseases in some countries.

A broad range of bacterial pathogens was detected, emphasizing the importance of conducting microbiological examinations and susceptibility testing to ensure appropriate antibiotic treatment.

Overall, a prevalence of 15.3% of diseases/clinical signs associated with the respiratory tract was observed during the study period. In contrast, previous studies focusing on emergency presentations noted lower occurrences of respiratory tract pathologies at 6.4% [15] and 12.6% [3] of the study population, respectively. High prevalences of mixed respiratory infections have also been identified as a common cause of mortality and illness in a prior study on mortality and morbidity of small poultry flocks, accounting for 21% of primary diagnoses [1]. This aligns with the understanding that multiple respiratory pathogens can concurrently affect the flock, contributing to multifactorial respiratory infections. The observation that mixed respiratory infections were a primary cause of clinical signs or death among small flocks, while unusual, is consistent with our findings.

In addition, various viral agents can affect the respiratory tract of chickens, while they are generally considered uncommon in backyard poultry [7,19]. In this survey, three chickens with lower respiratory tract diseases where diagnosed with IB (1.9%), and one with ILT (0.64%). The rarity of positive cases among suspicious instances aligns with findings from previous studies. It is important to note that in areas with endemic diseases, such as ILT in the United States, high prevalences could also exist in backyard poultry flocks [20]. Furthermore, veterinarians treating pet chickens should be aware that birds infected with mycoplasma, ILT, or IBV often encounter secondary gram-negative bacterial infections [1].

#### 4.5.3. Reproductive Tract

The second most prevalent set of diseases observed were reproductive tract pathologies in hens, also commonly seen in commercial layer flocks [21,22]. These pathologies are often multifactorial in origin [17]. While the ancestors of hens laid 1–2 clutches annually, commercial egg-layer breeds, producing approximately 301 eggs yearly, display non-seasonal laying behavior. This intense production predisposes them to reproductive issues. Additionally, infectious diseases, including IBV, MD, chronic stress-induced immunosuppression, and concurrent illnesses leading to secondary bacterial infections, notably *E. coli* ascending from the cloaca [23], contribute to infections of the reproductive tract. These infections commonly result in pathologies such as salpingitis, salpinx impaction, and egg yolk peritonitis. Testing affected chickens for underlying agents, such as IBV, could provide valuable insights into flock health, though it may not significantly influence treatment decisions for individual hens.

Reproductive tract diseases are frequently reported in backyard chickens, often presenting in older birds at the end of their peak laying performance. For example, a US survey reported a median age of 1.75 years for affected chickens [15], whereas in this study, the median age was 2.5 years (range: 0.4–6.4 years).

Treatment for reproductive tract diseases often requires surgery [24], successful procedures ensuring a good quality of life for the chickens [25]. Salpingohysterectomy is the most commonly reported surgical intervention, reflecting the commitment of backyard chicken owners to treat their chickens as pets rather than food sources. This trend is particularly notable in “rescued” hens from commercial flocks nearing the end of their productive lifespan [24]. However, salpingohysterectomy is a complex and costly procedure requiring detailed anatomical knowledge and surgical expertise [26]. Consequently, it is not routinely performed in general veterinary practices [15]. Referral to specialized clinics should be discussed with owners in such cases. For owners unable to afford surgery or when conservative management fails to resolve severe issues, such as oviduct impaction or egg-bound conditions, humane euthanasia must be considered as an ethical option.

#### 4.5.4. Skin, Feathers, and Appendages

Among the integument-related conditions observed, traumatic skin wounds accounted for nearly half of the cases. During the study period, a total prevalence of 10.3% was noted for trauma cases. Previous studies examining emergency presentations of backyard chickens highlighted an even higher prevalence of trauma cases [3,15]. Predation emerged as the leading cause of these injuries [5], a finding supported by our data. This highlights the importance of emergency veterinary services for pet chickens and underscores the need to advise owners to house their chickens in predator-proof enclosures.

In terms of outcomes, the overall prognosis for traumatic skin wounds was largely positive, with 16 out of 24 wounds healing without complications. However, many cases (15) required surgical intervention. Managing traumatic wounds in chickens follows similar principles to those used for dogs and cats, with adjustments made for the unique anatomical and physiological characteristics of poultry [15]. Despite this, some cases resulted in mortality: four chickens died, and four were euthanized. Notably, myiasis was associated with poor outcomes, contributing to four unfavorable prognoses.

Ectoparasites and pododermatitis constituted the second most common integument-related diseases observed. The overall prevalence of ectoparasites (3.6%) and pododermatitis (3.1%) was relatively low. Interestingly, surveys of backyard chicken owners frequently identify external parasites as the most common health issue affecting their flocks [4,5]. This discrepancy may arise because disease recognition can sometimes be challenging for owners. Ectoparasites are easily noticeable, and owners are generally more familiar with parasitic diseases compared to other infectious diseases [5].

Pododermatitis often results from husbandry deficiencies such as inadequate substrates, poor perches, underlying wounds, and obesity [17,27]. Addressing these cases requires implementing corrective measures focused on improving husbandry practices and environmental conditions. Recently, a novel approach using 3D-printed silicone shoes for treating pododermatitis in chickens has been described and has shown promising success [28].

#### 4.5.5. Locomotor System

Prevalence of clinical signs or diseases related to orthopedic issues was 10.7% (45/419). Of those, a final diagnosis could be determined in 28 cases. Malformations of the musculoskeletal system constituted the majority, followed by fractures, infectious or degenerative joint diseases, and only one case of femoral head necrosis. Fractures had the most favorable outcomes. Among those treated (3 with surgery and 4 with bandages), all recovered without complications. Fracture management in chickens generally follows guidelines used for small animals but requires specific adjustments. Bird bones heal faster than those of mammals and are more delicate, with many being pneumatized. Adequate calcium supplementation is particularly important for egg-laying chickens to ensure proper bone mineralization during healing.

Costly surgical procedures, such as bone plating, are often unnecessary. Most fractures can be successfully treated with bandages or less expensive techniques, such as intramedullary pinning with bandaging, external fixation, or tie-in fixations (a combination of intramedullary pinning and external fixation) [29].

#### 4.5.6. Infectious and Neoplastic Diseases

Marek’s disease is considered endemic in poultry populations [7]. While commercial poultry management has effectively controlled MD outbreaks through selective breeding and vaccination (administered in ovo or at day of hatch) [30], backyard poultry remain vulnerable. Vaccination in backyard flocks is less common, making MD a frequent cause of mortality [1,2,6]. Reported prevalences range from 11% to 27% in post-mortem studies [1,2,6,7,8].

In this study, MD was suspected in 33 cases (7.9%) among the 419 chickens presented at our clinic. Necropsy and histopathology were performed in 10 of these cases, all of which confirmed MD. Studies of emergency presentations report varying prevalences, including 7.7% [15] and 0.6% [3], respectively. MD manifests in various forms [31], typically affecting chickens between 10 and 20 weeks of age, though cases can occur outside this range. Of the confirmed cases in this study, eight chickens had known ages: four were younger than one year (2, 5, 7, and 9 months), and four were older than one year.

Veterinarians managing backyard chickens should maintain a high index of suspicion for MD. Once present in a flock, eradication is nearly impossible. The prognosis for individual chickens showing clinical signs is poor, though some may experience temporary improvement. Despite MD being confirmable only through post-mortem diagnostics, many owners choose to pursue treatment. Unfortunately, no specific treatment is available. Care typically involves anti-inflammatory medications along with supportive measures, such as fluid therapy, vitamin supplementation, force-feeding when necessary, and time.

Among the suspected MD cases in this study, eight chickens were lost to follow-up, nine were euthanized, two died, and four showed clinical improvement. Educating owners about MD is essential, particularly emphasizing the importance of acquiring vaccinated chickens to prevent outbreaks in new flocks. Such discussions are critical when MD is present in a flock, as many owners are unaware of the disease [5].

A comprehensive survey evaluating causes of mortality in backyard chickens in the United States [2] identified neoplasia or lymphoproliferative diseases as the most common primary diagnoses (42%) among deceased chickens. Diseases caused by avian leukosis virus (ALV) exhibit similar pathomorphological changes to MD, making differentiation challenging. In our study, only two chickens were confirmed to have ALV. However, this number may be underestimated, as not all euthanized or deceased chickens underwent necropsy. Additionally, some systemic neoplastic cases, such as carcinomatosis, may have involved underlying viral infections that went undetected.

While several zoonotic pathogens theoretically can be present in backyard chickens, none of the chickens in this survey showed suspicion or tested positive for avian influenza virus or Newcastle disease virus. Beyond these well-known viral zoonoses, various enteric bacteria have zoonotic potential, including *Salmonella enterica* and *Campylobacter* spp. Detection of these pathogens requires specific cultural procedures, which were performed on samples from the intestinal tract in this study. Indeed, *Campylobacter* spp. were detected in two chickens, though further species identification was not conducted.

Previous studies [1,16] have not strongly indicated that backyard poultry flocks act as significant reservoirs for these zoonotic diseases or pose substantial risks to public health [6]. However, it is worth noting that while rare, *Salmonella* spp. is supposed to be more common in small backyard flocks compared to the well-controlled commercial enterprises [17]. This may be attributed to the fact that backyard chickens often have outdoor access and free-range opportunities, increasing their contact with ubiquitous environmental bacteria. Conversely, a 2018 study found that *Salmonella* prevalence at the bird level in smallholder flocks was significantly lower than in federally inspected commercial flocks [32]. However, *Campylobacter* spp. showed higher prevalences in small poultry flocks compared to those reported for commercial broiler chickens from federal inspections [1]. There is no legal mandate to test smaller flocks for *Salmonella* or *Campylobacter* spp. Nevertheless, flock owners should be encouraged to assess the status of their chickens, especially if the eggs are intended for human consumption, to mitigate any potential zoonotic risks.

A common neoplastic condition observed in chickens is known as carcinomatosis, characterized by adenocarcinoma, primarily originating from the reproductive system. This condition typically presents as multifocal metastatic masses spread throughout the abdominal organs, often involving the gastrointestinal tract and sometimes accompanied by ascites [7,25,33]. In this study, carcinomatosis was the most prevalent neoplastic condition identified.

Spontaneous adenocarcinomas in the reproductive tract of laying hens are well-documented and represent a significant area of research, particularly because chickens serve as models for studying human ovarian adenocarcinomas [34,35]. This type of neoplasia becomes increasingly common as hens age [36], a trend reflected in our data. The high incidence of reproductive tract neoplasia, particularly in chickens older than two years, aligns with findings from previous studies [1,6].

Apart from reproductive tract neoplasia, a few other tumor types were identified, contributing to an overall neoplastic case incidence of 6.2%.

### 4.6. Treatment

Treatment was categorized based on the use of either licensed or off-label medications. Unfortunately, very few medications are licensed for use in egg-laying birds intended for human consumption [17]; there are no licensed antibiotics for treating bacterial infections of the urogenital tract, skin, or soft tissues in egg-laying chickens. Likewise, licensed anesthetics or analgesics for surgical procedures are unavailable. As a result, off-label medication use is common when treating pet chickens [3,15,24]. However, their use must be justified and should always adhere to local regulations [24]. Owners need to be educated, and the necessity and implications of using any off-label medication should be thoroughly discussed.

In this study, 355 prescribed medications were recorded, with 66.6% being used off-label. Antibiotics and analgesics were the most frequently prescribed off-label medications. Importantly, even if the owner declines consumption of a pet chicken, the bird is still legally considered a food-producing animal. An exception exists only for horses, rabbits kept as pets, and carrier pigeons, which are excluded from food production regulations and therefore face fewer restrictions on medicinal care. While many backyard chickens are kept for egg production, a considerable number are also considered pets [5]. Most owners choose to treat their chickens accordingly rather than consume their eggs. An official declaration designating pet chickens as non-food-producing animals would alleviate the discrepancy veterinarians face when treating them.

### 4.7. Outcome

Previous studies have reported poor survival rates after initial assessment among emergency presentations, with rates noted at 45.0% [15] and 57.4% [3], respectively. However, our data indicate higher survival rates, with 67.5% of cases surviving to discharge. As highlighted earlier, chickens are frequently brought in at advanced stages of illness, which becomes even more pronounced in emergency situations. This tendency might account for the elevated mortality rates, particularly in emergency presentations.

### 4.8. Owner Compliance

Most owners demonstrate a high level of compliance, as observed firsthand, when it comes to treating their individual chickens. Hospitalization was commonly required, and owners showed a willingness to continue with bandage changes, wound treatments, and administering medications at home and were open to regular necessary checkups. This enthusiasm and dedication to ensuring the well-being of the birds aligns with previous findings [5].

### 4.9. Preventive Care

Some diseases in backyard chickens stem from lapses in biosecurity management and deficiencies in husbandry. As with other animal species, educating owners before they acquire chickens is recommended or even mandated in some areas. Many common diseases can be at least partially prevented or alleviated through preventive medicine, combined with proper hygiene and husbandry practices [16]. In the commercial poultry sector, extensive efforts and strategies have been implemented to reduce and eliminate diseases [2]. In backyard poultry flocks, there is still room for improvement. Ideally, acquiring chickens from a single supplier would mitigate issues with infectious agents, but many flocks are mixed. If additional purchases cannot be avoided, a recommended precaution includes at least 30 days of quarantine away from other birds, accompanied by a health check of the newcomers during this time [14,17].

Vaccination stands as a crucial measure for maintaining the health of commercial poultry flocks; however, this practice is not commonly applied to backyard poultry flocks. While some chickens may have been vaccinated prior to acquisition [16], regular vaccine programs are not typically implemented for backyard chickens at present. None of the chickens observed during the study period had received any vaccines. Despite the potential benefits of targeted vaccination for specific diseases, various challenges hinder the implementation of effective vaccination programs in backyard flocks. First, many vaccines need to be administered to young or newly hatched chicks to be effective, reducing their utility in adult chickens [16]. Second, vaccines are usually available in quantities exceeding 1000 dosages and require frequent repetition to be effective, making their use economically unfeasible for many clients [14]. Third, in established “closed” flocks that do not introduce new birds or have contact with other flocks and have no history of prior diseases, vaccination can be considered unnecessary in most circumstances [16].

Regular parasitological examination of feces is also not commonly performed in backyard flocks. Educating owners on preventive management practices—such as quarantining new birds, conducting regular health checks, performing parasitological exams, maintaining hygiene, recognizing early signs of illness, and seeking timely veterinary care—could help reduce mortality rates and improve the overall health of backyard chickens.

This study has certain limitations that should be considered. First, the retrospective nature of data collection introduces inherent limitations, such as the potential for incomplete or missing records. Second, the types of presentations documented in this study may be influenced by the geographical distribution of the cases. Local factors, such as the distribution of endemic diseases and environmental conditions, can significantly impact the cases observed. Third, this study is biased toward chickens that were brought to specialized veterinary care, meaning it is not possible to provide an accurate estimate of the general prevalence of diseases in backyard chickens.

## 5. Conclusions

There is a noticeable demand for veterinary care among backyard chickens, reflecting their growing significance as companion animals, similar to more traditional pets. These chickens present with a wide range of disease conditions, often accompanied by nonspecific symptoms, making a thorough diagnostic workup essential in most cases. Most owners are eager to treat their chickens and seek advanced veterinary care, underscoring the need to officially classify backyard chickens as pets. This would help reduce restrictions on their medical care and exclude them from food production categories.

Owner compliance and education are critical in managing the health of these birds. Currently, there is a lack of disease prevention strategies in many backyard chicken flocks.

This study focused solely on chickens that sought specialized veterinary care. Further research into the health of entire flocks could provide deeper insights into the management of backyard chicken diseases, ultimately contributing to better care and welfare for these birds.

## Figures and Tables

**Table 1:** Admission causes, grouped according to their assigned system. For each animal, at least one admission cause was named by the owner, except for 33 of the cases where no admission cause was available in the medical records. n.a.—not applicable.

Assigned System	N (%)	Admission Cause	N (%)
Unspecific	240 (57.3)	Lethargy	127 (30.3)
		Anorexia	46 (11.0)
		Distended coelom	37 (8.8)
		Emaciation	17 (4.1)
		Mortality events	13 (3.1)
Respiratory tract	77 (18.4)	Respiratory distress	49 (11.7)
		nasal discharge	28 (6.7)
Locomotor	68 (16.2)	Lameness	33 (7.9)
		Inability to stand or to walk	24 (5.7)
		Leg/toe deformities	10 (2.4)
		Broken wing	1 (0.2)
Skin, feathers, and appendages	67 (16.0)	Skin lesions	23 (5.5)
		Disorders of skin and adnexa	18 (4.3)
		Feather damage	17 (4.1)
		Pododermatitis	9 (2.1)
Gastrointestinal tract	62 (14.8)	Diarrhea	26 (6.2)
		Enlargement of the crop	18 (4.3)
		Cloacal prolapse	14 (3.3)
		Regurgitating	4 (1.0)
Nervous system and eye	35 (8.4)	CNS symptoms	20 (4.8)
		Ocular discharge or swelling	15 (3.6)
Urogenital system	21 (5.0)	Occurrence of malformed eggs	13 (3.1)
		Cloacal tenesmus and discharge	8 (1.9)
n.a		Found on the street/lost animal	9 (2.1)
n.a		General health check	7 (1.7)

**Table 2:** Individual imaging diagnoses categorized by organ system and further arranged in alphabetical order made by the respective imaging modality (N = 130 imaging examinations), n.a. not applicable.

Assigned System	Imaging Diagnosis	CT	Ultrasonography	Radiography
Unspecific	Abdominal cyst	-	2	-
	ascites	1	34	1
	Soft tissue mass	4	4	1
	Splenomegaly	2	4	-
Respiratory tract	Focal bronchitis	1	-	-
	Lung infiltrations	3	-	-
	tracheitis	1	-	-
Locomotor	Pathologies of the skeletal system	6	-	8
Gastrointestinal tract	Abdominal hernia	-	2	-
	Gastric dilatation	1	-	1
	Hepatopathy	3	6	1
	Gastroenteritis	-	1	1
	Intestinal stenosis	-	-	1
	Peritonitis	-	12	-
Nervous system and eye	Blepharitis	-	1	-
urogenital system	Egg binding	1	15	-
	Egg material impaction	5	11	1
	Inhomogeneous ovary	-	1	-
	Malformed egg	-	17	-
	Renomegaly	3	-	-
	Salpingitis	8	17	-
	Salpingomegaly	-	-	2
n.a	Adipositas	1	-	-

**Table 3:** Isolated agent of a total of 87 samples taken from one or more regions—nose, conjunctiva, choana, trachea, pharynx, crop, cloaca, wounds, fluids, or inner organs. Six samples were negative for bacterial or fungal growth.

Isolated Pathogen	N
*E. coli*	41
*Pasteurella multocida*	19
Coagulase-negative *Staphylococcus* spp.	15
*Streptococcus* spp.	8
*Enterococcus* spp.	7
*Pseudomonas* spp.	6
*Gallibacterium anatis*	6
*Candida albicans*	6
*Clostridium* spp.	5
*Avibacterium* spp.	5
anaerobic bacteria	4
*Enterobacter* spp.	3
*Rothia nasimurium*	2
*Campylobacter* spp.	2
*Klebsiella* sp.	1
*Corynebacterium* sp.	1
*Acinetobacter* sp.	1
*Kasachstania telluris*	1

**Table 4:** *Mycoplasma* species identified in cases with either upper (URTI) or lower (LRTI) respiratory tract infection.

Mycoplasma Species	URTI	LRTI
*M. synoviae*	6	
*M. gallisepticum*	5	
*M. gallinarum*	4	1
*M. glycophilium*	4	
*M. pullorum*	4	
*Mycoplasma* sp. nov.	2	
*M. gallinaceum*	1	1
*M. lipofaciens*	1	

**Table 5:** Microorganisms isolated from specimens taken from the upper respiratory tract in cases where an upper respiratory tract infection was present.

Isolated Pathogen(s)	N	Isolated Pathogen(s)	N
*E. coli*	9	*Pseudomonas aeruginosa*	2
*Pasteurella multocida*	7	*Streptococcus pluranimalium*	1
Coagulase-neg. *Staphylococcus* spp.	6	*Streptococcus suis*	1
*Gallibacterium anatis*	2	*Acinetobacter* sp.	1
*Streptococcus* spp.	3	*Enterococcus cecorum*	1
*Proteus vulgaris*	2	*Enterococcus faecalis*	1
*Rothia nasimurium*	2	*Enterobacter* sp.	1
*Avibacterium paragallinarum*	2	*Candida albicans*	1

**Table 6:** Pathologies of the reproductive tract observed during the study period, categorized by the respective age of the cases. In 13 cases, the age was unknown.

Age (Years)	Egg Yolk Peritonitis/ Salpingitis/Salpinx Impaction	Neoplasia	Dystocia	Cloacal Prolapse	Shelled Eggs or Egg Material Freely Present in the Coelom	Soft-Shelled Eggs	Cyst Associated with the Salpinx	Sum
0.3–1	-	-	3	-	1	-	-	4
1–2	4	1	2	1	1	-	-	9
2–3	8	3	-	2	1	-	1	15
3–4	3	4	-	-	-	1	-	8
4–5	6	2	-	1	-	-	-	9
>5	3	-	-	-	1	-	-	4
Unknown	6	3	2	1	-	1	-	13
Sum	30	13	7	5	4	2	1	62

**Table 7:** Total numbers of the final diagnoses listed in descending order of occurrence. Treatments are categorized into surgical intervention, the use of only licensed medication, and the use of non-licensed medication; each case was assigned to one or more of these categories. The outcome of the respective cases is given in the following categories: improvement, lost to follow-up, recovery, died, or euthanized.

		Treatment			Outcome			
Final Diagnosis	N	Surgery	Licensed	Non- Licensed	Improved/Recovered	Lost	Died	Euthanized
Upper respiratory tract infection	50	1	36	29	25	23	1	1
Egg peritonitis	36	21	-	24	14	5	12	5
Soft tissue trauma	34	15	5	25	16	11	4	4
Endoparasitosis	31	-	21	11	9	17	1	4
Orthopedic issue	28	4	7	12	9	14	2	3
Neoplasia	26	14	1	19	1	2	9	14
Marek’s disease (suspected)	23	-	6	13	4	8	2	9
Marek’s disease (confirmed)	10	1	2	1	-	-	4	6
Pododermatitis	15	12	2	13	12	1	1	1
Ectoparasitosis	13	-	7	7	6	6	1	-
Coelomic mass	12	-	-	-	-	4	2	6
Mycoplasmosis	11	1	4	8	4	6	-	1
Cloacal prolapse	10	8	1	9	7	2	1	-
Infectious diseases	8	-	1	3	1	2	5	-
Dystocia	8	2	3	4	4	4	-	-
Salpingitis	7	2	1	4	1	3	1	2
Ophthalmic issue	7	1	3	4	4	3	-	-
Phytobezoar crop	7	6	1	7	3	2	2	-
Colibacillosis	6	2	1	3	-	-	5	1
Candidiasis	5	-	1	3	2	1	1	1
Crop dilatation	5	1	-	4	2	2	-	1
Gastroenteritis	4	-	1	3	2	1	-	1
Encephalitis	3	-	-	-	-	-	-	3
Soft shelled eggs	2	-	1	1	-	2	-	-
Pericholangitis	1	1	-	1	-	-	-	1
Beak deformities	1	-	-	-	1	-	-	-
Total (%)	100	25.3	28.9	57.3	35.0	32.8	14.9	17.6

**Table 8:** Surgical interventions (N = 92) undertaken in pet chickens (N = 363) within the study period.

Surgical Procedure	N
Salpingohysterectomy	24
Wound debridement	18
Laparotomy	13
Debriding pododermatitis lesions	10
Cloacal prolapse repositioning	7
Ingluviotomy	7
Cloacal egg development	3
Fracture repair	3
Mass resection	3
Sinus infraorbitalis sinusotomy	2
Air sac catheter	1
Ophthalmic surgery (coloboma)	1

**Table 9:** Overview of off-label used medication.

Medication Class	N
Antibiotic	194
Analgesic	164
Anesthetic	96
Antifungal	23
Antiparasitic	21
Other *	9

* ACE inhibitor, metoclopramide, GnRH analogue, corticosteroid.

## Data Availability

All data related to this study are presented in the manuscript.

## Supplementary Materials

The following supporting information can be downloaded at: https://www.mdpi.com/article/10.3390/ani15091288/s1, Table S1. Admission causes as reported by the owners (one or more causes assigned to each case), grouped according to their respective organ system including details of the corresponding diagnostic work-up performed based on the admission causes. It is important to note that there was no consensus on the diagnostic methods applied to each individual case. For instance, chickens presented with distended coelom underwent varying diagnostic procedures depending on the owner’s preferences and the specific circumstances of each case.
