# Supplementary material for: From Livestock to Companion: Admission Causes, Diagnostics, and Clinical Findings in Chickens Admitted to the Avian Clinic of the Vetmeduni Vienna, 2009–2019
Source: Animals (Basel). 2025 Apr 30;15(9):1288. doi: 10.3390/ani15091288 (PMC12071008; doi:10.3390/ani15091288)
Supplement: Supplementary file 1 [file animals-15-01288-s001.zip › animals-3573470-supplementary.pdf]

Supplementary Table S1. Admission causes as reported by the owners (one or more causes assigned to each case), grouped according to their respective organ system including details of the corresponding diagnostic work-up performed based on the admission causes. It is important to note that there was no consensus on the diagnostic methods applied to each individual case. For instance, chickens presented with distended coelom underwent varying diagnostic procedures depending on the owner's preferences and the specific circumstances of each case.

Assigned system	Admission cause (N)	Diagnostic imaging	Microbiological examination	Parasitological examination	Hematology	Serology	Necropsy/ Histopathology
unspecific	lethargy (127)	47	27	62	13	-	23
	anorexia (46)	22	10	17	4	-	10
	distended coelom (37)	31	2	7	-	-	10
	emaciation (17)	4	3	11	-	-	6
	mortality events (13)	-	4	6	-	-	7
respiratory tract	respiratory distress (49)	9	23	18	4	3	8
	nasal discharge (28)	4	20	12	2	3	4
locomotor	lameness (33)	9	3	12	-	-	1
	inability to stand or to walk (24)	10	3	9	1	-	7
	leg/ toe deformities (10)	2	-	-	-	-	-
	broken wing (1)	-	-	-	-	-	-
skin, feathers and appendages	skin lesions (23)	4	2	5	-	-	2
	disorders of skin and adnexa (18)	2	3	10	-	-	3
	feather damage (17)	1	5	8	2	1	-
	pododermatitis (9)	-	-	1	-	-	-
gastrointestinal tract	diarrhea (26)	10	9	9	4	-	5
	enlargement of the crop (18)	6	8	11	2	-	4
	cloacal prolapse (14)	9	-	6	1	-	1
	regurgitating (4)	1	1	-	-	-	2
nervous system and eye	CNS symptoms (20)	4	2	11	2	-	3
	ocular discharge or swelling (15)	1	8	2	-	-	1
urogenital system	occurrence of malformed eggs (15)	8	1	3	1	-	-
	cloacal tenesmus and discharge (8)e	6	1	3	-	-	-
n.a	found on the street/lost animal (9)	1	-	2	-	-	-
n.a	general health check (7)	-	1	2	-	1	-
